# Twenty-Eight Years of Invasive Meningococcal Disease Surveillance in the Autonomous Province of Vojvodina, Serbia: Epidemiological Trends and Implications for Enhanced Surveillance and Vaccination Policy

**DOI:** 10.3390/vaccines13090945

**Published:** 2025-09-03

**Authors:** Mioljub Ristić, Vladimir Vuković, Tatjana Pustahija, Snežana Medić, Gorana Dragovac, Vladimir Petrović

**Affiliations:** 1 Institute of Public Health of Vojvodina, 21000 Novi Sad, Serbia; tatjana.pustahija@mf.uns.ac.rs (T.P.); snezana.medic@mf.uns.ac.rs (S.M.); gorana.dragovac@mf.uns.ac.rs (G.D.); vladimir.petrovic@mf.uns.ac.rs (V.P.); 2 Department of Epidemiology, Faculty of Medicine, University of Novi Sad, 21000 Novi Sad, Serbia

**Keywords:** invasive meningococcal disease, surveillance, incidence rate trends, age-specific patterns, seasonality, mortality, case fatality rate, Serbia, AP Vojvodina

## Abstract

Background/Objectives: Meningococcal disease (MD) remains a significant public health concern worldwide. In Serbia, mandatory immunization against MD with the meningococcal polysaccharide vaccine (MenAC) for high-risk groups and international travelers was introduced in 2006. Since 2017, the polysaccharide vaccine has been replaced with the quadrivalent meningococcal conjugate vaccine (MenACWY). The aim of this study was to analyze long-term trends in incidence, age-specific patterns, seasonality, and lethality of invasive meningococcal disease (IMD) in the Autonomous Province of Vojvodina (AP Vojvodina), Serbia, over a 28-year period. Methods: A descriptive study analyzed all reported cases of IMD in AP Vojvodina, from 1997 to 2024. Data were obtained from the regional communicable disease surveillance system, based on mandatory hospital reporting and case classification according to national and WHO guidelines. Temporal, demographic, and clinical characteristics, along with disease outcomes, were analyzed. Results: From 1997 to 2024, 175 IMD cases were reported in AP Vojvodina. The annual incidence peaked in 1997 (1.24/100,000), with smaller surges in 2003 and 2005. Since 2006, coinciding with the introduction of immunization against MD, a sustained decline has been observed, with incidence rarely exceeding 0.30/100,000. A slight resurgence occurred in 2023–2024, with 13 cases reported. From 1997 to 2024, IMD in AP Vojvodina exhibited a clear seasonal pattern, with most cases occurring in winter and early spring, peaking in January (17%), March (12%), and February (11%), and the fewest cases occuring in the summer months. Throughout the study period, the highest IMD incidence rates were consistently observed among infants <1 year of age and children aged 1–4 years, with peaks of up to 22.9/100,000 and 16.0/100,000, respectively. Incidence was much lower in older age groups, especially adults. After a 2006 peak, rates declined across all ages, with a slight resurgence in 2023–2024 among children and adolescents. Children aged 1–4 years made up the largest share of IMD cases, peaking in January–March (45.1%). Half of the infant cases were recorded in October–November, while cases in older children, adolescents, and adults were fewer and showed varied monthly patterns, with small peaks in winter and early spring. During the 28-year study period, the highest IMD mortality rate was observed among infants <1 year of age (0.59 per 100,000 population), followed by children aged 1–4 years (0.32 per 100,000). Mortality rates declined progressively with increasing age, with the lowest rate recorded among individuals aged ≥40 years (0.01 per 100,000). Of the 175 IMD cases reported in AP Vojvodina (1997–2024), 21 were fatal (case fatality rate [CFR] = 12.0%). The CFR of IMD varied across age groups. The highest CFR was observed among individuals aged ≥40 years (21.4%), followed by the 5–9 years (17.4%) and <1 year (16.7%) age groups. None of the patients had been vaccinated against MD. Fatal outcomes were more common in children aged 1–4 years and among rural residents, though differences were not statistically significant (*p* > 0.05). Most deaths (57.1%) occurred in the first quarter of the year. A strong association was found between clinical form and outcome, with meningococcal sepsis being significantly more frequently associated with fatality than meningitis (*p* = 0.0002). Deaths were sporadic over time, with most occurring within 1–2 days of notification. All confirmed fatal cases were due to serogroup B. Conclusions: MD remains a rare yet serious public health threat in AP Vojvodina. Mortality rates indicate that the public health impact of this disease is greatest among the youngest age groups; however, the risk of death, i.e., disease severity, does not appear to be age dependent. The recent rise in cases, high fatality among sepsis patients, and absence of prior vaccination among all IMD cases highlight the need for enhanced surveillance, physician education, and consideration of introducing both MenACWY and MenB vaccines for high-risk groups.

## 1. Introduction

Invasive meningococcal disease (IMD), caused by infection with Neisseria meningitidis (*N. meningitidis*) bacteria, remains a significant public health concern worldwide. IMD most frequently presents as meningitis, septicemia, or a combination of both, while less common clinical manifestations include pneumonia, septic arthritis, and pericarditis [1,2]. The disease typically has a sudden onset, with meningitis or sepsis developing within 24 h, and can lead to severe complications or death if not promptly treated [1,2,3,4]. The case fatality rate (CFR) of IMD is approximately 10%, and among survivors, 10–20% experience long-term, disabling sequelae [1,2,3]. Although the introduction of meningococcal vaccines has led to a marked decline in incidence in many countries, small outbreaks and fatal cases continue to be reported, especially in areas with suboptimal vaccine coverage or delayed implementation of immunization programs [5,6,7].

In the European region, the burden of IMD remains unevenly distributed, with infants, young children, and adolescents representing the most affected age groups [8]. Seasonal peaks are typically observed in late winter and early spring [8,9]. CFRs vary depending on age, serogroup distribution, and access to healthcare settings [1,2,8,10].

In accordance with the Regulations on the Immunization Program of the Population against Certain Infectious Diseases in the Republic of Serbia in force at the time, a decision to introduce the polysaccharide meningococcal vaccine was adopted in 2002. Its implementation, however, was delayed until 2006. Under this regulation, the bivalent meningococcal polysaccharide vaccine (MenAC) was indicated, based on clinical criteria, for patients aged ≥2 years with anatomical or functional asplenia, for those with complement (C5–C9) deficiencies, as well as for international travelers [11].

In 2017, a new regulation introduced the quadrivalent meningococcal conjugate vaccine (MenACWY) for mandatory immunization individuals with anatomical or functional asplenia (e.g., splenectomy, sickle cell anemia), complement deficiencies (C5–C9), or those undergoing hematopoietic stem cell transplantation. Vaccination also became mandatory for laboratory personnel exposed to *N. meningitidis* cultures with potential for aerosol generation, and for close contacts of confirmed meningococcal disease (MD) cases. It was further required for pilgrims traveling to Saudi Arabia for Hajj, and for individuals in transit through or residing in endemic areas for extended periods. In addition, vaccination was recommended, though not mandatory, for students and pupils residing in dormitories, as well as for military conscripts. The Menactra vaccine (MenACWY) was used for individuals aged ≥2 years [12]. In 2019, indication was revised to specify the dosing schedule: for children aged 9–23 months, two doses were required, and for those aged 2–55 years, a single dose was sufficient [13]. Since 2025, Menactra has been replaced by MenQuadfi (MenACWY), following the 2017 indications but approved for individuals aged ≥12 months [14].

The aim of this paper was to provide a comprehensive epidemiological overview of MD in the Autonomous Province of Vojvodina (AP Vojvodina), from 1997 to 2024, focusing on incidence trends, age-specific patterns, seasonality, and clinical outcomes.

## 2. Materials and Methods

### 2.1. Study Design, Data Sources, and Variables

This descriptive study was conducted in AP Vojvodina, located in northern Serbia, with a population declining from 2,031,992 in 2002 to 1,729,606 in 2022 [15]. Data were obtained from the regional communicable disease surveillance system, managed by the Institute of Public Health of Vojvodina (IPHV), Novi Sad. All reported cases of IMD from 1997 to 2024 were included. Notifications of IMD cases were submitted by hospitals across AP Vojvodina through the national mandatory reporting system. Case classification followed the national surveillance standards and the World Health Organization (WHO) guidelines [16]:

Confirmed case: *N. meningitidis* identified by culture or polymerase chain reaction (PCR) from a purpuric skin lesion or any normally sterile site (e.g., blood, cerebrospinal fluid [CSF], synovial fluid).

Probable (clinical) case: Clinical diagnosis of meningitis or septicemia, plus at least one of the following criteria:

- Presence of a purpuric rash with *N. meningitidis* considered the most likely cause (linked to a confirmed case, with other causes of hemorrhagic rash excluded or deemed less likely);
- Detection of Gram-negative diplococci in a normally sterile site (blood, CSF) or in a purpuric skin lesion;
- Detection of *N. meningitidis* antigen (e.g., by latex agglutination) from a normally sterile site or a purpuric skin lesion.

### 2.2. Statistical Analysis

Demographic (gender, age, place of residence, district of AP Vojvodina), temporal (month, year), and clinical (clinical presentation, outcome) variables were analyzed. Incidence rates per 100,000 population were calculated using annual population estimates [15]. Age-specific mortality rates were calculated using average population estimates [15] by age group and expressed per 100,000 inhabitants. These rates were then divided by 28 to account for the 28-year observation period. Seasonal distribution was assessed by calendar month. Group comparisons (e.g., survivors vs. fatalities) were performed using Pearson’s chi-square or Fisher’s exact test, as appropriate. A *p*-value of <0.05 was considered statistically significant.

All annual data were officially reported to the Center for Disease Control and Prevention at the IPHV, as part of the routine communicable disease surveillance system in the province. Surveillance activities were conducted in collaboration with the district Institutes of Public Health located in the administrative centers of the seven districts of AP Vojvodina: Subotica (North Bačka), Sombor (West Bačka), Novi Sad (South Bačka), Kikinda (North Banat), Zrenjanin (Central Banat), Pančevo (South Banat), and Sremska Mitrovica (Srem).

### 2.3. Ethical Considerations

The data from this retrospective study are based on the results of IMD surveillance conducted between 1997 and 2024 as part of the routine surveillance at the Center for Disease Control and Prevention, IPHV, Novi Sad; therefore, approval from the Ethics Committee was not required in Serbia.

## 3. Results

### 3.1. Trends and Seasonal Patterns

From 1997 to 2024, a total of 175 cases of IMD were reported in AP Vojvodina. The annual number of cases varied considerably, ranging from 0 (2011, 2018, and 2020–2022) to 25 in 1997, when the highest annual incidence (1.24 per 100,000 inhabitants) was recorded. A notable increase was also recorded in 2003 (18 cases; 0.89/100,000) and 2005 (20 cases; 0.98/100,000).

**Figure 1:**
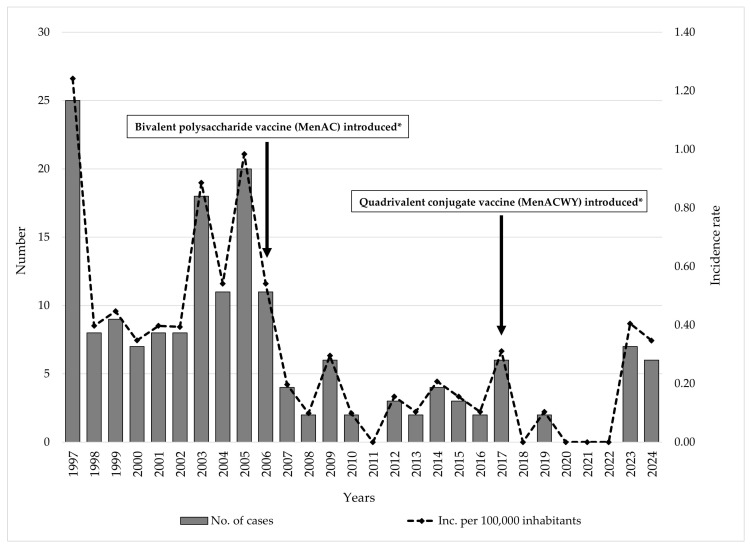
Trend of meningococcal disease and timeline of vaccine introduction against meningococcal disease in AP Vojvodina, Serbia, 1997–2024. Legend: * Mandatory for high-risk groups and international travelers.

Following the introduction of mandatory immunization against MD for high-risk groups in 2006, both the number of cases and incidence rates declined markedly, with annual incidence rarely exceeding 0.30 per 100,000 inhabitants. In recent years, a slight resurgence was observed, with 7 cases (0.40/100,000) reported in 2023 and 6 cases (0.35/100,000) in 2024 (Figure 1).

**Figure 2:**
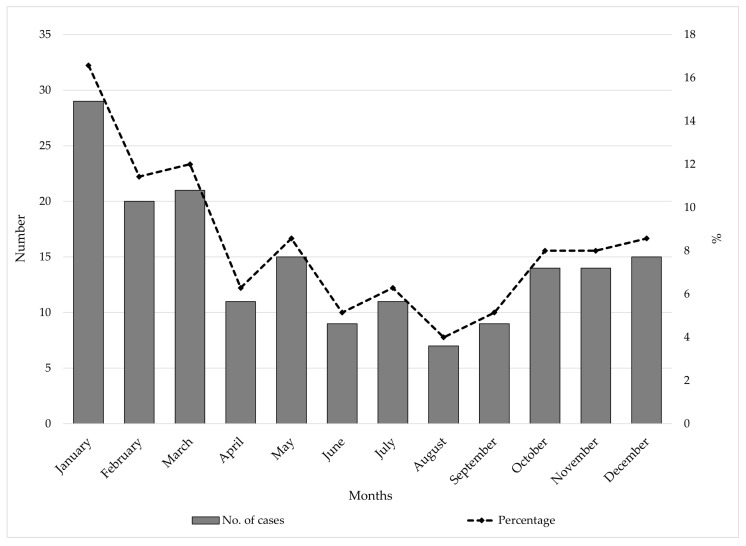
Seasonal distribution of meningococcal disease in AP Vojvodina, Serbia, 1997–2024.

From 1997 to 2024, IMD in AP Vojvodina showed a seasonal pattern, with most cases reported in January (17%), March (12%), and February (11%). The lowest percentage of cases were observed during the summer months, particularly in August (4%), June and September (5% each), suggesting a winter-spring peak in registered cases (Figure 2).

### 3.2. Age-Specific Distribution

**Figure 3:**
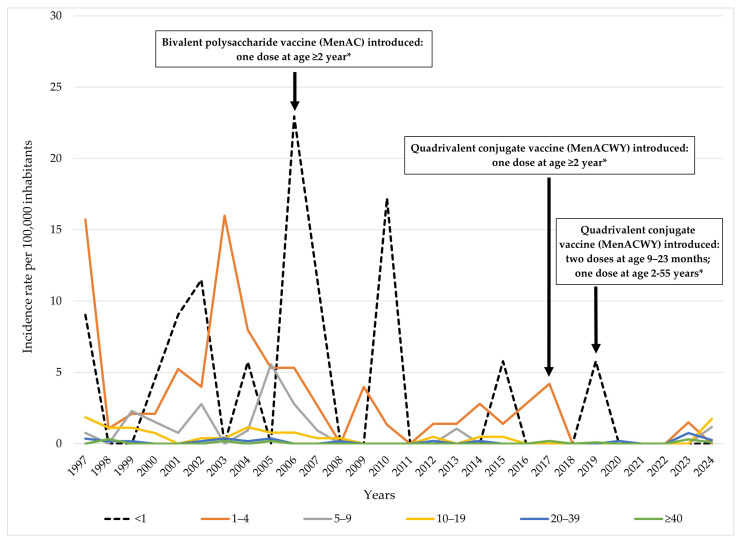
Age-specific incidence rates of meningococcal disease and timeline of vaccine introduction by target age groups in AP Vojvodina, Serbia, 1997–2024. Legend: * Mandatory for high-risk groups and international travelers.

During the observed period, the highest incidence rates of IMD were consistently recorded in infants <1 year of age and in children aged 1–4 years. Notably, in 2006, the incidence among infants peaked at 22.9 per 100,000, while in 1997 and 2003, children aged 1–4 years had rates of 15.7 and 16.0 per 100,000, respectively. Other significant peaks among infants were observed in 2001 (9.0/100,000), 2002 and 2007 (11.5/100,000 in each year), and 2010 (17.2/100,000). In children aged 5–9 years, incidence rates remained relatively low, with sporadic increases, in 1999 (2.3), 2002 (2.8), 2005 (5.6), and 2006 (2.8/100,000). Among adolescents aged 10–19 years, rates were generally low, with values not exceeding 2.0 throughout the study period, the highest being 1.9 in 1997 and 1.7 in 2024. The adult population (20–39 years and ≥40 years) showed consistently low incidence rates throughout the entire period, typically below 0.5 per 100,000, with occasional isolated increases. In the ≥40 age group, the disease was rarely reported, with values reaching 0.3 per 100,000 only in a few years (1998 and 2023). Following a peak in 2006, when vaccination against MD was introduced for high-risk groups and international travelers aged ≥2 years, incidence declined sharply in subsequent years across all age groups except infants. A further decline in the incidence of IMD among the youngest age group was observed after 2019, following the introduction of vaccination for individuals aged 9 months to 55 years. However, in recent years (2023–2024), a slight resurgence has been noted, particularly among children aged 1–4 and 5–9 years, as well as adolescents aged 10–19 years (Figure 3).

**Figure 4:**
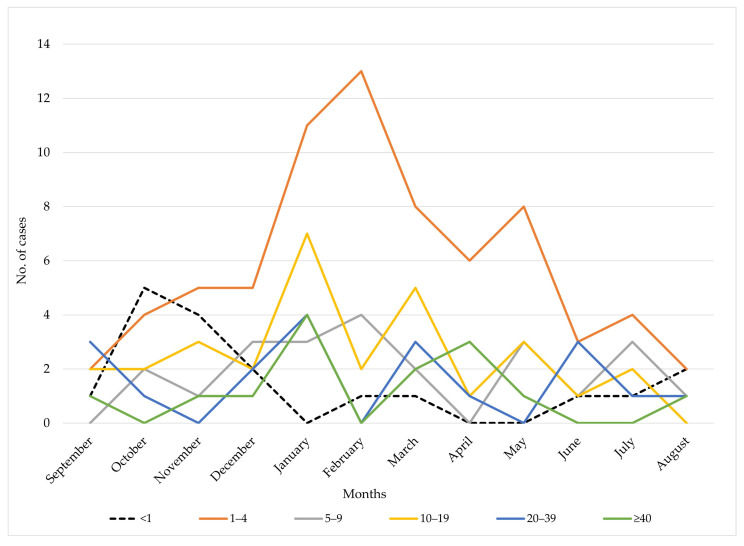
Monthly distribution of meningococcal disease cases by age group in AP Vojvodina, 1997–2024.

During the study period, infants <1 year of age (*n* = 18) had most cases in October (*n* = 5; 27.8%) and November (*n* = 4; 22.2%). Children aged 1–4 years accounted for the largest share of cases most months, peaking in January (*n* = 11; 15.5%), February (*n* = 13; 18.3%), and March (*n* = 8; 11.3%) out of 71 total cases. Cases among 5–9-year-olds (*n* = 23) were relatively stable year-round, with a slight peak in February (*n* = 4; 17.4%). The 10–19 age group (*n* = 30) peaked in January (*n* = 7; 23.3%) and March (*n* = 5; 16.7%). Adults 20–39 years (*n* = 19) were most affected in January (*n* = 4; 21.1%) and March (*n* = 3; 15.8%). Patients aged ≥40 years (*n* = 14) had highest case counts in January (*n* = 4; 28.6%) and April (*n* = 3; 21.4%), with no cases reported in February, June, July, or October (Figure 4).

### 3.3. Mortality and Case Fatality Rates

During the 28-year observation period, the highest IMD mortality rate was observed among infants <1 year of age (0.59 per 100,000 population), followed by children aged 1–4 years (0.32 per 100,000). Mortality rates declined progressively with increasing age, with the lowest rate recorded among individuals aged ≥40 years (0.01 per 100,000).

**Figure 5:**
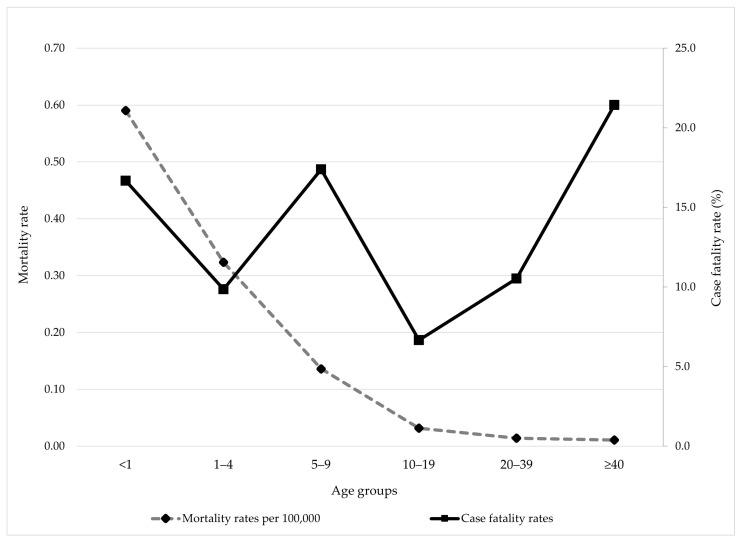
Mortality and case fatality rate by age groups of meningococcal disease in AP Vojvodina, 1997–2024.

The CFR of IMD varied across age groups. The highest CFR was observed among individuals aged ≥40 years (21.4%), followed by the 5–9 years (17.4%) and <1 year (16.7%) age groups (Figure 5).

### 3.4. Clinical Outcomes

Out of all reported IMD cases in AP Vojvodina during the study period, 21 patients died (CFR = 12.0%). None of the patients had been previously vaccinated against MD.

The gender distribution was not significantly different between the outcome groups (*p* = 0.5524), with males comprising 57.1% of survivors and 66.7% of those who died. Fatal outcomes were more frequently observed among children aged 1–4 years (33.3%), but differences among age groups were not statistically significant (*p* = 0.6480). Among survivors, the majority (41.6%) were aged 1–4 years, followed by the 10–19 (18.2%) age group.

More deaths occurred among residents of rural areas (52.4%) compared with those from urban areas (47.6%); however, this difference was not statistically significant when compared with IMD patients from urban areas (*p* = 0.3793).

Among the 154 survivors, the highest proportion was from the South Bačka district (48.7%), followed by West Bačka (18.8%), and Srem (11.7%). Similarly, among the 21 fatal cases, South Bačka also had the highest share (38.1%), followed by North Bačka and West Bačka (both 19.1%). No fatal cases were recorded in the North Banat district, while one death each was reported in Central Banat and South Banat (4.8%, respectively). The proportion of deaths in North Bačka (19.1%) was notably higher than its share among survivors (7.8%), whereas South Bačka showed a relatively lower share of deaths compared to its proportion among survivors. However, the overall distribution of fatal outcomes by district was not statistically significant (*p* = 0.6513).

The highest proportion of fatal cases was recorded in the first quarter of the year (January–March), accounting for 57.1% of deaths compared with 37.7% of survivors in the same period; however, the difference by quarter of the year was not statistically significant (*p* = 0.3368).

A significant association was observed between clinical presentation and outcome (*p* = 0.0002). Among those who died, 76.2% had meningococcal sepsis, while only 23.8% had meningococcal meningitis (Table 1).

Over the 28-year period, fatal meningococcal cases occurred sporadically, with no consistent temporal pattern. Notably, four deaths were recorded in both 1997 and 2005, representing the years with the highest annual fatal burden. Fatal cases were observed across both pediatric and adult age groups, including three infants <1 year of age and three individuals aged ≥40 years. The time interval between disease notification and death was typically short. In 15 of 21 cases (71.4%), death occurred within 1 to 2 days following notification. Patients with a prolonged clinical course (≥10 days) were aged between 35 and 61 years. The majority of deaths (15/21; 71.4%) were laboratory-confirmed, while six cases were classified based on clinical criteria alone. Fatal outcomes were recorded throughout the year but were most frequently registered in February and March (5 cases each), followed by November (2 cases), suggesting a seasonal trend toward late winter and early spring. The most recent fatal case was reported in January 2024 (Table 2). All laboratory-confirmed fatal cases were of serogroup B.

## 4. Discussion

Our 28-year retrospective analysis offers important insights into the long-term epidemiological patterns of IMD in AP Vojvodina, Serbia, with a focus on age-specific incidence rates, seasonal distribution, and clinical outcomes. Although the overall burden of IMD markedly declined after the mid-2000s, the observed recent resurgence underscores the importance of sustained surveillance and the potential need to reevaluate and strengthen preventive strategies, including vaccination policies.

### 4.1. Temporal Trends

A recent study by Findlow et al. [17] demonstrated the profound impact of the COVID-19 pandemic on IMD epidemiology across multiple countries. The widespread implementation of non-pharmaceutical interventions, including lockdowns, school closures, travel restrictions, and physical distancing, led to a marked and unprecedented reduction in IMD incidence, particularly among children and adolescents, who are key drivers of meningococcal transmission dynamics. Following the relaxation of these measures, heterogeneous patterns of IMD resurgence were observed across countries and serogroups, with some regions reporting a rebound of cases as early as mid-to-late 2022. Notably, shifts in age distribution toward adolescents and young adults, as well as an increased proportion of serogroup B cases, were documented. Findings from the mentioned study [17] highlight the potential for immunity gaps in cohorts who missed routine vaccinations or were not exposed to meningococcal carriage during the pandemic, which may increase the risk of outbreaks in the coming years. In line with this, sustained surveillance and robust routine and catch-up immunization programs remain essential to mitigate these risks and maintain population-level protection against IMD [1,2,4,8,17].

According to the most recent Annual Epidemiological Report by the European Centre for Disease Prevention and Control (ECDC) [8], the incidence of IMD across the European Union/European Economic Area (EU/EEA) remained historically low in 2021, largely due to sustained COVID-19 control measures. However, a marked resurgence was documented in 2022, with 2292 confirmed cases—representing a 104% increase compared to 2021.

In South-Eastern Europe, pre-pandemic notification rates (2010–2019) ranged from 0.13 per 100,000 annually in Serbia to 0.82 per 100,000 in Croatia, with Serbia consistently reporting the lowest rates, which may, in part, reflect underreporting. During 2020, IMD incidence sharply decreased across the region, coinciding with the widespread implementation of lockdowns and social distancing measures [18].

Globally, IMD incidence varies substantially, from 0.3 to 4 per 100,000 in the Americas and 0.2–14 per 100,000 in Europe, to as high as 1000 per 100,000 during epidemics in the African meningitis belt [19].

In our study, the highest IMD incidence in AP Vojvodina was recorded in 1997, with additional smaller peaks in 2003 and 2005, consistent with outbreak periods reported in other Central and Eastern European countries during the 1990s and early 2000s [18,19]. Following 2006, incidence declined markedly and remained low for over a decade. Although data on immunization coverage against MD are unavailable for AP Vojvodina, this declining trend is unlikely to be attributable solely to vaccination, as routine meningococcal immunization remains limited to high-risk groups and travelers [11,12,13,14,20], warranting further investigation. Nevertheless, a modest increase in cases during 2023–2024, is noteworthy. While such fluctuations may partly reflect the stochastic nature of small populations, the re-emergence of cases in previously less-affected age groups could indicate increased transmission, waning population immunity, or historical underreporting. Similar post-pandemic resurgences have been documented in other countries with low vaccine coverage [17], underscoring the importance of enhanced surveillance and consideration of broader immunization strategies.

### 4.2. Age-Specific Patterns

Across the EU/EEA, a post-pandemic rebound of IMD incidence was particularly pronounced in 2022 among children under 5 years of age, especially infants, who consistently experience the highest rates. Increased incidence was also noted among adolescents and young adults (15–24 years), reflecting a return to pre-pandemic transmission dynamics [8]. According to the study by Tzanakaki et al. [18], in 2019—the most recent year unaffected by COVID-19—the highest age-standardized IMD incidence rates in the region were reported among infants (<1 year of age) in the Czech Republic, Greece, Hungary, Poland, and Romania, while such data were not available for Croatia, Serbia, and Ukraine, which were also included in the study. Children aged 1–4 years represented the second most affected group, followed by adolescents and young adults, except in Slovenia.

Consistent with these regional and global patterns [1,8,17,18], our findings demonstrate a substantial burden of IMD among the youngest children, with sporadic cases in older pediatric age groups and rare occurrences in adults. Specifically, infants <1 year of age and children aged 1–4 years in AP Vojvodina bear the greatest burden, with most cases occurring during the winter months. The modest resurgence observed in 2023–2024 in this age group, although partly attributable to the stochastic variation in small populations, underscores the need to sustain targeted vaccination strategies, which in Serbia remain limited to high-risk cohorts.

### 4.3. Seasonal Variation

This age-stratified monthly overview suggests temporal clustering within certain age groups, particularly among young children in winter and early spring months. The seasonal pattern observed in AP Vojvodina, with a pronounced winter–spring peak (January–March), aligns with global IMD epidemiology, likely driven by factors such as indoor crowding, concurrent respiratory infections, and reduced mucosal immunity during colder months [8,9,21]. Notably, a few infant cases also occurred in autumn, which may reflect sporadic individual exposures rather than broader transmission dynamics.

### 4.4. Case Fatality Rates

Results of the mentioned study in the nine South-Eastern European countries [18] showed that CFRs remained generally stable across the 2010–2020 period in each country, although notable inter-country differences were observed. Higher CFRs were reported in Hungary, ranging from 9.4% in 2014 to 25.7% in 2015. In contrast, lower CFRs were recorded in Croatia (2.8% in 2015 to 10.0% in 2016), Greece (0% in 2011 and 2020 to 11.8% in 2018), and Poland (3.8% in 2020 to 13.5% in 2019). During this period, between 0 and 2 deaths per year were registered in Serbia, and the annual CFRs appear low, but this was probably due to underreporting, as the data for this study were collected from the Health Statistical Yearbook of the Republic of Serbia rather than from the National Institute of Public Health.

According to the latest ECDC report (2022) [8], the CFR of IMD in Europe was 10%, consistent with WHO estimates [1]. A recent meta-analysis of 40 studies published between 2000 and 2018, encompassing 163,758 IMD patients, reported CFRs ranging from 4.1% to 20.0%, with a pooled estimate of 8.3% [22]. We found an overall CFR of 12.0%, which falls within the expected global range of 10–15% for IMD [22,23]. Our findings suggest that the highest mortality rate was observed among infants (0.59 per 100,000 population), with a steady decline across older age groups, reaching the lowest rate among individuals aged ≥40 years (0.01 per 100,000). However, the CFRs demonstrated a bimodal distribution, with the highest CFRs recorded among infants (16.7%) and the oldest age groups (21.4%), consistent with previous comprehensive systematic reviews and meta-analyses showing that older adults tend to experience higher CFRs than younger populations [22]. Although children aged 1–4 years in our study accounted for the highest number of deaths, no statistically significant differences in CFR were observed across age groups, indicating that the probability of death was relatively evenly distributed. Notably, clinical presentation was strongly associated with outcome: meningococcal sepsis accounted for over three-quarters of all fatalities and was significantly more frequent among fatal cases compared with meningitis. These findings underscore the fulminant nature of meningococcemia and the critical need for early recognition and aggressive management. Despite advances in treatment, a significant proportion of pediatric IMD survivors experience long-term sequelae, emphasizing the substantial morbidity burden of the disease [24,25].

We found that the number of both survived and fatal cases was highest in the South Bačka District of AP Vojvodina. This pattern may, in part, reflect the fact that the South Bačka District hosts the University Clinical Center of Vojvodina—the only tertiary-level healthcare facility in the AP Vojvodina—which likely receives the most severe and complex cases from across the region. In addition, South Bačka is the most populous district in AP Vojvodina with 607,454 inhabitants in 2022 (i.e., 18.7% of the province’s population) [15], which may further contribute to the higher number of reported IMD cases in this area.

Although not statistically significant, the higher number of deaths in rural compared to urban settings is epidemiologically relevant. Factors such as limited access to care, differences in health-seeking behavior, and diagnostic delays may contribute to this disparity, as noted in other low- and middle-income contexts and highlighted by the Global Meningococcal Initiative [19,26]. The clustering of deaths in late winter and early spring further underscores the need for heightened clinical vigilance during these peak months. We also found that most fatalities occurred within 48 h of disease notification, reflecting the rapid clinical progression typical of meningococcal sepsis, as consistently reported in previous studies [23,25,27].

### 4.5. Global, Regional, and Local Serogroup Distribution and Vaccination Policies

A review of the global epidemiology of IMD from 2010 to 2019 indicates that its incidence remains low but varies considerably across regions, with infants, young children, adolescents, and, in some settings, older adults being the most affected. Serogroups B, C, W, and Y predominate, with notable increases in W and Y in several countries, while serogroup A has largely disappeared from Africa following vaccine introduction. The authors of this analysis highlighted that the unpredictable and dynamic epidemiology underscores the need for sustained surveillance and regionally adapted vaccination strategies [4].

According to the most recent ECDC report, the majority of IMD cases in EU/EEA countries, with a documented serogroup in 2022, belonged to serogroup B (62%), followed by serogroups Y (16%), W (10%), and C (6%). Serogroup B predominated across all age groups below 65 years [8]. The global distribution of the different serogroups of *N. meningitidis* may change over time. Historically, in the United States, serogroup B has accounted for approximately 40% of IMD cases, while serogroups C, W, Y, and nongroupable (non-encapsulated) meningococci contributed smaller proportions. However, during 2022 and 2023, a marked shift was observed, with serogroup Y driving the overall increase in incidence and representing a growing proportion of reported cases [2].

Although serogroup distribution was unavailable in our dataset, except for fatal cases, previous study covering the 2010–2020 period indicate that in Serbia, serogroup B was the predominant cause of IMD between 2010 and 2017, whereas a relative increase in cases caused by serogroups C and Y was documented from 2018 to 2020 [18]. Furthermore, data from the Serbian National Reference Laboratory for Meningococcus (Sombor, West Bačka District, Serbia) revealed that among 53 invasive *N. meningitidis* isolates collected from children under 18 years of age between January 2009 and January 2018, serogroup B predominated, accounting for over 80% of isolates. Serogroup C accounted for 11.3%, while serogroups W135 and Y were detected in only two (3.8%) and one (1.9%) isolates, respectively [28].

Taken together, these findings underscore critical considerations for regional vaccination strategies, particularly in light of the dynamic serogroup distribution observed across Europe and the United States [2,8,18]. The authors of the aforementioned studies also emphasize substantial gaps in MD surveillance and underreporting in several countries, while noting concerning epidemiological trends in nations with more consistent reporting systems. This policy gap—exacerbated by limited public awareness and restricted access to vaccines—may leave vulnerable populations, including infants, adolescents, and individuals with underlying conditions, at heightened risk for IMD.

These observations call for an evidence-based reassessment of national vaccination policies in South-Eastern Europe. Specifically, the inclusion of MenB and MenACWY vaccines should be considered, with prioritization for high-risk groups and, potentially, extension toward broader age-based immunization strategies [18].

By the end of July 2025, meningococcal vaccination was recommended in 19 EU/EEA countries, with the exception of Bulgaria, Croatia, Denmark, Estonia, Finland, Latvia, Norway, Romania, Slovenia, and Sweden. France was the only country where meningococcal vaccination was mandatory, following a schedule of three doses during the first year of life (two doses of MenB and one dose of MenACWY), and one booster dose in the second year (either MenB or MenACWY) [29]. France introduced mandatory childhood immunization against MenC as early as 2018. In this country, vaccination against MenB has been recommended since 2022, and, from 2025 onwards, the national schedule has followed the mandatory scheme described above [29]. These policy decisions were informed by several factors, including genotyping data of IMD strains circulating between 1975 and 2022 [30], seroepidemiological studies assessing population-level immunity to specific *N. meningitidis* serogroups [31], and the incidence and characteristics of IMD observed in the post-COVID-19 period in France [17].

In Serbia, meningococcal vaccines are not included in the routine national immunization schedule for the general population; since 2017, the quadrivalent MenACWY vaccine has been available only to vulnerable population groups and international travelers [11,12,13,14]. The most recent amendments to the recommendations, issued in January 2025, maintained these indications but expanded eligibility to include individuals aged ≥12 months [14].

In light of the continued occurrence of sporadic cases, the associated mortality, and the particular susceptibility of infants and young children, these findings support consideration of targeted vaccination strategies, particularly for infants, young children, and adolescents. Notably, previous studies [18,28] indicate that serogroup B has been the most common cause of IMD in Serbia, with the majority of cases occurring among children aged 0–4 years. In addition, all laboratory-confirmed fatal IMD cases in the study period in AP Vojvodina were caused by serogroup B. Following the initial licensure of the four-component, protein-based MenB vaccine (4CMenB) in the European Union in 2013, several countries introduced recommendations for its use in high-risk groups and, more recently, implemented routine infant immunization programs. Evidence from these countries demonstrates a substantial decline in IMD incidence following the introduction of such measures [32]. Furthermore, several European countries, as well as the United States, have adopted MenB and/or MenACWY vaccination programs in recent years, achieving measurable reductions in IMD incidence [32,33,34]. Since all laboratory-confirmed fatal IMD cases in our study were caused by serogroup B, there is a strong rationale for introducing a protein-based MenB vaccine in our territory and for promoting a change in the current non-vaccination practice, given that none of the fatal cases had been previously vaccinated.

### 4.6. Strengths and Limitations

This study has several strengths. First, it covers a long 28-year observation period, enabling the analysis of temporal trends, seasonal patterns, and age-specific variations in IMD incidence and mortality. Second, it is based on a population-wide, legally mandated surveillance system with standardized case definitions aligned with WHO guidelines, ensuring comparability over time. Third, the inclusion of both confirmed and probably (clinical) cases allowed for a more complete assessment of the disease burden, particularly in earlier years when diagnostic capacity was limited. Fourth, detailed stratification by age, month, clinical presentation, and outcome provided granular insights into the epidemiological profile of IMD in AP Vojvodina.

Nevertheless, several limitations should be acknowledged. First, potential underreporting or misclassification of cases cannot be excluded, especially for atypical presentations. Second, changes in diagnostic techniques and reporting accuracy (for which we have no data) over nearly three decades may have influenced case detection and classification. Third, complete data on serogroup distribution, underlying medical conditions (comorbidities) among IMD cases, and immunization coverage among high-risk groups and international travelers across the entire territory of AP Vojvodina were lacking, limiting the interpretation of epidemiological trends and the vaccine-prevention potential.

## 5. Conclusions

MD remains a serious, albeit rare, public health threat in AP Vojvodina. Mortality rates indicate that its impact is greatest among the youngest age groups; however, the risk of death, and thus disease severity, does not appear to be age dependent. While the overall incidence has steadily declined since the mid-2000s, a recent modest increase in cases—particularly among children—underscores the importance of continued surveillance and risk assessment. All laboratory-confirmed fatal IMD cases reported during the 28-year period in AP Vojvodina were caused by serogroup B. The high CFR among patients with meningococcal sepsis highlights the urgency of early clinical recognition and supports the possible introduction of meningococcal vaccination for high-risk groups in Serbia, including not only ACWY-containing vaccines but also those targeting serogroup B-related IMD. Given that none of the patients in this study had received prior vaccination against MD, enhancing physician awareness and education at all levels of healthcare is a critical priority for improving vaccine uptake and disease prevention. Continuous surveillance of IMD cases and circulating serogroups, along with achieving high vaccination coverage—at least among high-risk groups—is crucial for evaluating the impact of prevention strategies and guiding future public health decisions. Most of the aforementioned efforts are also recommended by the WHO, which issued a global call in 2021 to eliminate meningitis caused by *N. meningitidis*, *Streptococcus pneumoniae*, *Haemophilus influenzae*, and *Streptococcus agalactiae* (group B) by 2030 [35]. Finally, the interdisciplinary relevance of IMD data extends beyond medicine and epidemiology, offering valuable insights for mathematical modeling of vaccine effectiveness and durability and for shaping future prevention strategies.

## Figures and Tables

**Table 1:** Comparison of demographic and clinical characteristics of patients with meningococcal disease in AP Vojvodina, 1997–2024, by outcome.

Variable		Survived (*n* = 154)		Fatal Cases (*n* = 21)		*p*-Value
		*n*	%	*n*	%	
Gender	Male	88	57.14	14	66.67	0.5524
	Female	66	42.86	7	33.33	
Age group (years)	<1	15	9.74	3	14.29	0.6480
	1–4	64	41.56	7	33.33	
	5–9	19	12.34	4	19.05	
	10–19	28	18.18	2	9.52	
	20–39	17	11.04	2	9.52	
	≥40	11	7.14	3	14.29	
Place of residence	Urban	93	60.39	10	47.62	0.3793
	Rural	61	39.61	11	52.38	
District of the AP Vojvodina	North Bačka	12	7.79	4	19.05	0.6513
	West Bačka	29	18.83	4	19.05	
	South Bačka	75	48.70	8	38.10	
	North Banat	7	4.55	0	0.00	
	Central Banat	8	5.19	1	4.76	
	South Banat	5	3.25	1	4.76	
	Srem	18	11.69	3	14.29	
Quarter of the year	January–March	58	37.66	12	57.14	0.3368
	April–June	33	21.43	2	9.52	
	July–September	24	15.58	3	14.29	
	October–December	39	25.32	4	19.05	
Diagnosis	Meningococcal meningitis	105	68.18	5	23.81	0.0002
	Meningococcal sepsis	49	31.82	16	76.19	

**Table 2:** Characteristics of meningococcal disease cases with fatal outcome in AP Vojvodina, 1997–2024.

Patient	Year of Death	Gender	Age (in Years)	Area of Residence	Diagnosis	Days Between Disease Notification and Exitus Letalis	Classification	Month of Death
1	1997	Female	1	Urban	MM	2	CC	February
2	1997	Female	4	Urban	MM	1	LCC	March
3	1997	Female	35	Rural	MM	11	LCC	April
4	1997	Male	1	Urban	MS	4	CC	May
5	1998	Male	61	Rural	MM	10	LCC	September
6	1999	Female	7	Rural	MS	1	CC	March
7	2001	Male	2	Rural	MS	1	LCC	February
8	2001	Male	7	Urban	MS	2	LCC	October
9	2002	Female	24	Urban	MS	6	LCC	February
10	2002	Male	9	Urban	MS	1	CC	July
11	2003	Male	1	Rural	MS	1	LCC	March
12	2003	Male	49	Rural	MS	18	LCC	June
13	2003	Male	1	Rural	MS	2	LCC	November
14	2005	Male	1	Rural	MS	1	CC	February
15	2005	Female	7	Urban	MS	1	LCC	July
16	2005	Male	61	Urban	MM	10	LCC	November
17	2005	Male	<1	Rural	MS	2	CC	February
18	2006	Male	<1	Rural	MS	1	LCC	October
19	2007	Male	17	Rural	MS	1	LCC	March
20	2009	Female	<1	Urban	MS	2	LCC	March
21	2024	Male	14	Urban	MS	2	LCC	January

Legend: MM—Meningococcal meningitis; MS—Meningococcal sepsis; CC—Clinical case; LCC—Laboratory-confirmed case.

## Data Availability

The data that support the findings of this study are available from the corresponding author upon reasonable request.
